# Performance and Enhanced Efficiency Induced by Cold Plasma on SAPO-34 Membranes for CO_2_ and CH_4_ Mixtures

**DOI:** 10.3390/membranes14080178

**Published:** 2024-08-20

**Authors:** Fnu Gorky, Vashanti Storr, Grace Jones, Apolo Nambo, Jacek B. Jasinski, Maria L. Carreon

**Affiliations:** 1 Ralph E. Martin Department of Chemical Engineering, University of Arkansas, Fayetteville, AR 72701, USA; gorky@uark.edu (F.G.); vstorr@uark.edu (V.S.); gej006@uark.edu (G.J.); 2 Bert Thin Films, LLC., 625 Myrtle St, Louisville, KY 40298, USA; apolo@bertthinfilms.com; 3 Conn Center for Renewable Energy Research, University of Louisville, Louisville, KY 40292, USA; jacek.jasinski@louisville.edu

**Keywords:** non-thermal plasma separation, sapo-34 membranes, CO_2_ and CH_4_ mixtures, electrification

## Abstract

In this study, we investigate the influence of cold-plasma-induced enhanced performance and efficiency of SAPO-34 membranes in the separation of CO_2_ and CH_4_ mixtures. Placing the herein presented research in a broader context, we aim to address the question of whether cold plasma can significantly impact the membrane performance. We subjected SAPO-34 membranes to plasma mild disturbances and analyzed their performance in separating CO_2_ and CH_4_. Our findings reveal a notable enhancement in membrane efficiency and sustained performance when exposed to cold plasma. The pulsed plasma separation displayed improved structural integrity, and the experimental results indicated that the linear structure of CO₂ facilitates the distortion of electron clouds in response to the electric field, a property known as polarizability, which aids in effective separation. Plausible mechanistic insight indicated that the intermolecular forces facilitated an integral role in SAPO-34 membranes exhibiting strong electrostatic interactions. In conclusion, our research highlights the potential of cold plasma as a promising technique for improving the performance of SAPO-34 membranes in gas mixtures at atmospheric pressures, providing valuable insights for optimizing membrane technology in carbon capture and gas separation applications.

## 1. Introduction

Chemical separations contribute a significant share of industrial energy consumption in the United States, amounting to approximately 50%. Moreover, separation processes contribute up to 15% of the total energy consumption in the country [1]. Many separation techniques used in industrial processes, such as distillation, evaporation and drying, rely on thermal energy as a driving force. These thermally driven separation methods can be energy intensive. Alternative separation technologies without heat could make chemical separations more energy efficient, such as membrane separation [2,3]. Specifically in natural gas wells, carbon dioxide constitutes an undesirable impurity; hence, its removal is required due to its detrimental effects attributed to its acidic and corrosive properties when exposed to water [4,5]. Amine absorption is a conventional technique where liquid amine solutions selectively absorb CO_2_ [6,7,8]. Membrane separation utilizes specialized membranes to selectively permeate CH_4_ while blocking CO_2_ [9,10,11,12]. Pressure swing adsorption (PSA) exploits the differential adsorption capacities of gases on solid adsorbents [13,14], while cryogenic distillation relies on the different boiling points of CO_2_ and CH_4_ at low temperatures [15,16]. Among various methods, amine absorption is a commonly used technique for separating CO_2_ and CH_4_ in natural gas processing [8]. However, this technique is susceptible to corrosion [17], requiring corrosion-resistant materials [18] and constant maintenance. Furthermore, the energy-intensive regeneration process contributes to higher operational costs [19]. Similarly, cryogenic distillation for CO_2_ and CH_4_ separation is an energy-intensive process, since it requires compression and cooling, leading to increased operational costs [20]. The complex equipment and large footprint, combined with challenges in scalability and maintenance of cryogenic distillation units involving extreme temperatures, can pose technical challenges and can make this method less economical and practical for some applications. On the other hand, pressure swing adsorption (PSA) for CO_2_ and CH_4_ separation faces challenges with additional heat management and cooling within the unit leading to energy consumption, impacting overall efficiency. As an alternative, membrane separation offers advantages such as reduced energy consumption. In general, membrane-based processes are approximately 20% less energy intensive than cryogenic distillation [20]. The compact design and lower maintenance make membrane-based separation an appealing option for overcoming some of the drawbacks associated with other technologies for CO_2_–CH_4_ separation [21]. The implementation of advanced membrane technologies for CO_2_–CH_4_ separation holds the potential to mitigate costs associated with natural gas purification.

The integration of membranes made from porous crystals with uniform micropores is particularly attractive for various industrially relevant gas separation applications, such as single gas permeation and gas mixture separations of H_2_, CH_4_, N_2_ and CO_2_ using zeolitic membranes [22,23,24]; separations of H_2_, CH_4_, N_2_, O_2_ and CO_2_ using metal–organic frameworks [10,25,26]; and separations of H_2_, CH_4_, N_2_, O_2_, CO_2_, Kr and Xe using porous organic cages [27,28]. Specifically, zeolites are noted for their highly desirable properties, including uniform micro-sized pores, their high surface area that offers enhanced gas adsorption capacity and being chemically and thermally stable, which can be advantageous for selective gas separation, such as H_2_/CH_4_, CO_2_/N_2_, CO_2_/CH_4_, N_2_/CH_4_, H_2_/N_2_ [13,24,29,30,31,32,33]. Presently, gas gathering compression is commonly applied to surge gas pressure from natural gas wellhead pressure to approximately 1015–1450 Psi for transport to a gas processing plant or sales pipeline [34]. The literature indicates that membranes are operated and designed for handling such high pressures [35].

Pairing non-thermal-plasma (NTP) and membrane separation is specifically mentioned as a promising approach to reduce the energy consumption for process intensification [36,37,38]. Therefore, in this work, we focus on membrane separation performance using NTP.

NTP has been reported to influence the surface interaction and materials’ properties in a wide range of applications [39,40,41,42]. Specifically, interest toward carbon capture and membranes with NTP has been widely described in the literature [43,44]. Recently, it has been reported that by tuning the ionization, crystal restructuring can be possible by adjusting the electron beam energy and dose rate, offering potential development for applications in treating ceramics, improving thin film quality and precision engineering of nanostructures in oxide materials with octahedral or tetrahedral building blocks [45].

However, the current challenge when designing separation systems that can be paired with certain plasma sources involves the separation of gases at sub-atmospheric pressures. Currently, the designs for plasma-based reactors or separation systems for decarbonization processes are generally intended for low to atmospheric pressures. Hence, this work aims to address the challenges of gas separation at sub-atmospheric pressures. A previous work by our group explored the effects of plasma-induced desorption of CO_2_ and CH_4_ on various SAPOs [46].

The global shift from fossil-based to renewable-based energy systems requires significant advancements across various energy sectors [47]. The current chemical separation processes are highly energy intensive and require substantial improvements to facilitate this transition. Specifically, adsorption-based separation processes hold the potential for full electrification through innovative strategies involving the development of stimuli-responsive adsorbents and swing processes. Several efforts have been conducted in the literature for process intensification [48,49]. Despite their potential, the electrification of adsorptive separation presents formidable challenges [50]. The primary initial challenge consists of finding efficient and cost-effective methods for providing and managing the required electrical energy to realize the benefits of reduced energy consumption at both small and large scales [50]. Electrification and carbon capture technologies are pivotal for achieving net-zero emissions in the chemical sector. Electrifying adsorptive separation processes requires substantial electrical energy, which may raise costs and environmental issues, especially if generated from non-renewable energy. Currently, 63.3% of global electricity is fossil-fuel-based, while wind and solar contribute 5.3% and 2.7%, respectively [50]. This dependence on non-renewables challenges the sustainability, environmental impact and economic feasibility of such processes. Addressing these issues is vital for advancing sustainable and decarbonized technologies. Decarbonizing the global industry to net-zero emissions by 2050–2070 is crucial for climate stabilization, requiring supply-side technologies like carbon capture and electrification [51].

Furthermore, this work focuses on capturing and separating greenhouse gases (GHGs) at sub-atmospheric pressures. While achieving effective separations at atmospheric pressures can be ambitious due to the requirement of high pressures to achieve separation, we aim to overcome this limitation by integrating intermittent *electrification* to enhance the separation process. In this work, our main hypothesis aims to show an enhancement in solubility and diffusivity by induced polarization of CO_2_ through an electric disturbance rather than through chemical modification of the surface on an *in-house*-built SAPO-34 membrane-plasma separation system. At the same time, we aim to understand the plausible effect of an intermittent plasma dosage on membranes for performance longevity along with its possible effect on the gases’ selectivity at sub-atmospheric pressures. Herein, we present the use of microporous crystalline SAPO-34 membranes coupled with non-thermal plasma pulses as a plausible selective separation process for CO_2_/CH_4_ gases.

## 2. Materials and Methods

### 2.1. Membrane Synthesis

#### 2.1.1. SAPO-34 Seed Synthesis

The synthesis method was adapted from elsewhere [9]. SAPO-34 seeds were synthesized with a molar ratio 1.0 Al_2_O_3_:2.0 P_2_O_5_:0.6 SiO_2_:4.0 TEAOH:150 H_2_O. At room temperature, first, aluminum isopropoxide was added to DI water and stirred for 1 h, followed by adding phosphoric acid dropwise and stirring for 2 h, followed by adding the silica source (Ludox AS-40 silica) and stirring for 3 h, finally adding tetraethylammonium hydroxide (TEAOH) and allowing to stir at room temperature overnight. The mother gel was then transferred to a Teflon autoclave and treated at 180 °C for 7 h, followed by centrifuging the gel at 3500 rpm for 12 min to afford SAPO-34 crystals. They were dried at 100 °C overnight before usage.

#### 2.1.2. SAPO-34 Membrane Synthesis

SAPO-34 membranes were prepared using the secondary seeded growth method inside the surface of porous α-Al_2_O_3_ tubes. The 6 cm supports (Inopor GmbH, Veilsdorf, Germany) have an inside diameter of 0.7 cm and an outside diameter of 1.1 cm and are asymmetric within the inner layer, which has a pore size of 100 nm. The effective support area was ∼7.0–7.5 cm^2^. Initially, two bare cylindrical alumina supports were seeded with pre-prepared seeds.

Upon receiving the alumina tubes, both ends were glazed. The effective permeation area is ~7.5 cm^2^. The glazed supports were calcined at 950 °C for 15 min with heating and cooling rates of 1 °C/min, treated twice in boiling water for 30 min and dried at 150 °C overnight. The composition of the SAPO-34 membrane gel was as follows: 1.0 Al_2_O_3_:2.0 P_2_O_5_:0.6SiO_2_:1.0 TEAOH:1.6 dipropylamine (DPA):150 or 300 H_2_O. The membrane gel was synthesized similarly to seeds, but, after adding dipropylamine, the solution was aged for 4 days at 50 °C while being stirred continuously.

The tubular supports were seeded by rubbing SAPO-34 seeds with a cotton swab onto the inner surface. The outer surface of the supports was wrapped with Teflon tape to prevent membrane growth on the outside surface. Multiple layers of seeding were necessary to allow even nucleation. The seeded supports were placed inside a Teflon liner and were filled with membrane gel (entire support was immersed in the gel) to be hydrothermally treated at 230 °C for 6 h in an autoclave. After cooling and synthesis, the supports were washed slowly with DI water and dried at 100 °C. To calcine the membrane, a heating rate and cooling rate of 0.8 °C/min and a temperature of 400 °C (held for 4 min) were used.

#### 2.1.3. Membrane Continuity

Before testing the membranes for separation performance, the synthesized SAPO-34 membranes were tested for continuity. For this purpose, a 1.8% propane/nitrogen gas mixture was passed through the membranes for 60 min. Based on experimental data (please refer to Figure S1), the feed pressures were kept constant at 40 psi, controlled by the cylinder’s delivery pressure. The retentate stream pressures were maintained at 15.3 psi after closing the retentate stream with a needle valve. The permeate stream data were collected for 60 min with a total flow rate of 200 mL/min of the 1.8% propane/nitrogen gas mixture. The gases were tracked using gas chromatography (GC) for accuracy. Since these experiments were conducted at sub-atmospheric pressures, the gas collections were carried out in gas bags for all streams to ensure accurate data collection, performed in triplicate. Based on the continuity testing results, the pressures remained steady with no fluctuations during experimental data collection, indicating consistent performance and the presence of a continuous membrane. Furthermore, the permeate stream successfully separated propane from the feed and retentate, confirming the presence of a continuous membrane.

### 2.2. Plasma Membrane System Design

**Figure 1:**
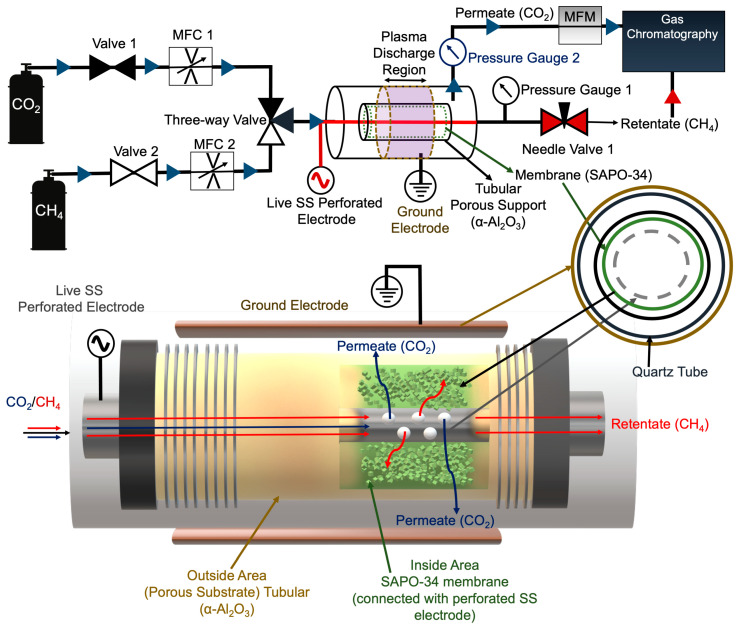
Schematics of plasma-catalytic membrane separation of CO_2_/CH_4_ mixtures.

The detailed plasma separation system is shown in Figure 1. We synthesized SAPO-34 membranes grown on porous alumina tubular supports, as described before. In this setup, the porous alumina cylindrical support is connected through stainless-steel fittings to a perforated stainless-steel tube that helps to support and transport the gas mixture and acts as a live electrode. The stainless-steel fittings and the outer membrane support were sandwiched between the quartz tube, a dielectric, and on top of the quartz tube, three stainless steel (SS) adapters acted as ground electrodes for creating the plasma discharge. The design of the separation unit exhibits a double dielectric discharge barrier, generating plasma both inside and outside the membrane. In this setup, the live electrode is connected to a perforated electrode, generating plasma at the interface inside the membrane with the perforated electrode and outside the membrane with an inner copper mesh. The ground electrode consists of an outer copper mesh located outside the quartz tube completing the circuit. The total flow rate was set at 200 sccm; the retentate pressure (inside the membrane support) was 10 psi; and the permeate pressure (outside the membrane) was 0 Psi. In this work, the gas flow rates were measured through mass flow controllers and mixed via a 3-way valve connected to the separator inlet. The methane and carbon dioxide were fed constantly at ratios of 92:8 and 50:50 (CH_4_:CO_2_). Pressure gradients within (retentate) and outside the membrane (permeate) were monitored using pressure gauges. The permeate pressure was controlled using a needle valve; during operations, the needle valve was closed, allowing the retentate stream to pressurize. The pressures were controlled using the input from the delivery pressure gauge of the cylinder. Furthermore, the permeate stream (outside the membrane) was connected to a mass flow meter to accurately monitor the flow rate. Retentate and permeate streams consisted of independent pressure gauges for actively monitoring the pressure gradient. According to the experimental data, when the retentate stream was pressurized at 10 Psi, the permeate stream pressure was observed below 0.2 Psi. Data collection consisted of each cycle running for 25 min. Both retentate and permeate streams were collected by opening and closing the needle valve quickly until the pressure gauge displayed 0 Psi; this was done to avoid contamination from the original feed pressure. The gases exiting were collected in gas bags for both retentate and permeate streams and further injected manually in GC for quantification. Gas monitoring and analysis were conducted using an Agilent 8860 gas chromatograph, equipped with an HP-PLOTU column (30 m × 0.32 mm × 10 μm) and hydrogen gas as the carrier, as described in Figure 1 and Figure S2. The plasma membrane separation system was improved from our previous work, where the selective gas separation was limited [52,53].

CO_2_/CH_4_ separation was quantified by measuring gas permeability. Permeability is commonly used for comparing the gas separation performance of different materials (see Equation (1)).

(1) $$\mathrm{Permeability}\;(P)=\frac{flux\;(J)}{pressure\;drop(∆p)/thickness(l)}$$

The permeability (P) of a gas can be defined as the product of the diffusivity coefficient (D) and solubility coefficient (S) of a gas in the membrane material and expressed as P = D × S. The solubility coefficient of a gas represents the ratio of the concentration of gas adsorbed or dissolved in the membrane at equilibrium to the gas pressure. Diffusivity is the mobility of the gas molecules in the membrane as they diffuse through the membranes. It is well noted that solubility represents a thermodynamic property, and diffusivity is a kinetic property of the gases. The membrane is expected to exhibit either high diffusivity, high solubility or both to achieve high permeabilities. Diffusivity usually depends on the kinetic diameter of gas molecules, and solubility depends on the polarity and chemistry of the membrane materials. The solubility coefficient of a gas depends on the interaction with the membrane material. Functional groups on the materials, such as hydroxyl and amine, interact with polar gases, such as CO_2_, resulting in increased solubility and an increase in gas permeability.

For the CO_2_/CH_4_ separation experiments, the retentate pressures were consistently maintained for different feed ratios, and the permeate pressure was measured to be > 0.2 Psi (minimum detection on the pressure gauge). Feed pressures were controlled using the cylinder regulator; the flow rates were controlled using a mass flow controller (MFC) set at a constant 200 sccm (92:8 and 50:50 CH_4_:CO_2_) stream, further verified by monitoring the permeate flow using a mass flow meter (MFM) at the exit. The experimental design involved testing with no plasma, constant plasma and pulsed plasma. The experiments were initiated with the “no plasma” operations conducted without plasma with pristine SAPO-34 membrane to compare the separation performances. Constant plasma operations were conducted at constant low power (~0.6 Watts). We demonstrate the effect with and without plasma on SAPO-34 membrane morphology in the upcoming section. Finally, we conducted pulsed plasma experiments involving short plasma dosage (~5 min) and no plasma for 20 min during a 25 min run with a collection time of 5 min for gases’ quantification.

### 2.3. Membrane Characterization

The scanning electron microscopy (SEM) images were obtained using a Philips XL30 ESEM (Eindhoven, The Netherlands) with a field-emission source at low pressure, with an acceleration voltage of 30 kV (please see Figure S3). Raman spectroscopy was conducted using a Renishaw InVia Raman Microscope Leica DM 2500M (Wotton-under-Edge, Gloucestershire, UK) with LASER 785 nm and a Transmission Electron Microscope (TEM) Tecnai G2 F20-TWIN (TF20) from FEI (Hillsboro, OR, USA) with a field-emission source at low pressure, with an acceleration voltage of 100 kV. The X-ray photoelectron spectroscopy (XPS) measurements were conducted using a VG Scientific MultiLab 3000 (Sussex, UK) ultra-high vacuum system and an Al Kα X-ray source (hν ≈ 1486.6 eV) at a base pressure in the 10^–9^ Torr range and the VersaProbe station from Physical Electronics (PHI) with a scanning X-ray monochromator (Chanhassen, MN, USA). XPS spectra were collected at an electron emission angle of 54.7° relative to the surface normal. For each sample, in addition to a low-resolution survey spectrum from 1200 to 0 eV, high-resolution spectra of C1s, O1s, P2p, Si2p and Al2p were also collected. High-resolution spectra were analyzed using XPSPEAK4.1 software, with the Shirley function used for background fitting and Gaussian line shape for peak deconvolution. The elemental composition of these samples was also evaluated based on energy-dispersive X-ray spectroscopy (EDS) analysis combined with transmission electron microscopy (TEM). These measurements were conducted in a 200 kV FEI Talos TEM (Hillsboro, OR, USA). TEM samples were prepared by scratching off some of the membranes and dispersing them on copper-grid-supported holey carbon films.

## 3. Results and Discussion

### 3.1. CO_2_ and CH_4_ Permeance

Before conducting the separation experiments with carbon dioxide/methane mixtures, to verify the diffusivity of gas molecules in binary mixtures, we conducted breakthrough experiments at sub-atmospheric pressure (~10 psi) for a 50:50 nitrogen/methane gas mixture using a SAPO-34 membrane. Studying breakthrough experiments on CH_4_/N_2_ in a membrane separation study for CO_2_/CH_4_ aids in understanding membrane selectivity and its performance. According to the experimental data (please refer to Figure S5), nitrogen displayed faster breakthrough compared to methane. This observation correlates with findings from other membranes reported in the literature [54,55]. The data verified that the diffusivity of nitrogen (3.6 Å) was favored over the slightly larger methane molecule (3.8 Å) on the SAPO-34 membrane, which had a minimum pore size of 3.8 Å. Furthermore, the contrast in diffusivities between nitrogen and methane proved to be more critical than competitive adsorption effects.

**Figure 2:**
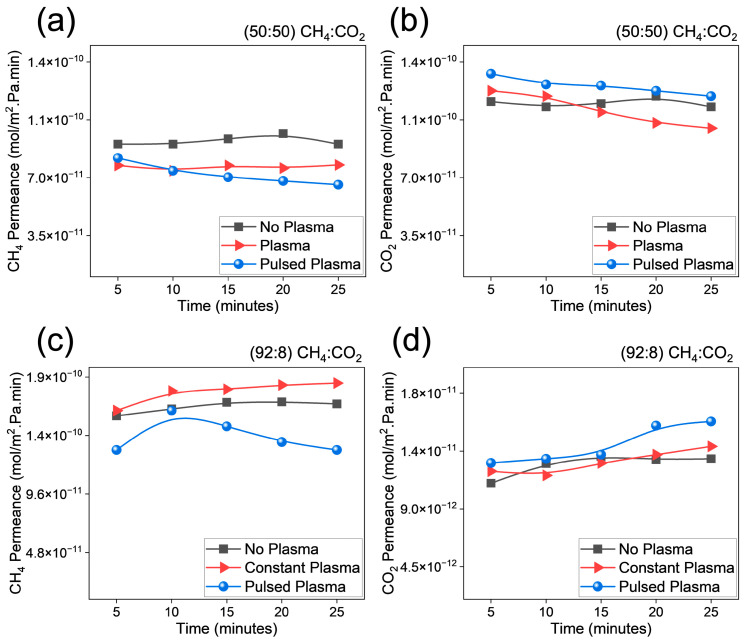
Analysis of permeance ((mol)/(m^2^·s·Pa)) at total flow rate of 200 sccm and different feed ratios. (**a**) Methane and (**b**) carbon dioxide with feed ratio 50:50 (CH_4_:CO_2_); (**c**) methane and (**d**) carbon dioxide with feed ratio 92:8 (CH_4_:CO_2_). Note: Y-axis is different for part (**d**) due to low CO_2_ permeance.

Based on the experimental data, after each cycle (occurring every 25 min), the experimental data displayed a decline in performance over time. Table 1 provides a comparative analysis of CO_2_/CH_4_ separation performance with and without plasma treatment at a feed ratio of 92:8 and 50:50 (CH_4_:CO_2_) over a period of 25 min. The performance metrics assessment included CO_2_ and CH_4_ permeance, selectivity (α), permeate pressure and the separation index (π). At a feed ratio of 50:50, CO_2_ permeance was higher, particularly in the pulsed plasma mode, which showed an increased CO_2_ permeance of 1.2 × 10^−10^ (mol)/(m^2^·s·Pa). CH_4_ permeance was lower compared to the 92:8 ratio, with the pulsed plasma mode exhibiting the lowest value (6.6 × 10^−11^ (mol)/(m^2^·s·Pa)). Selectivity was significantly improved in the pulsed plasma mode (1.81) compared to the other modes (1.25–1.29). The separation performance decreased with time, as similar trends were observed in the literature, particularly at higher pressures [12]. The separation index was markedly higher in the pulsed plasma mode (1.3 × 10^−7^), indicating enhanced separation efficiency and making it the most effective operation for CO_2_/CH_4_ separation at this ratio, suggesting that pulsed plasma enhances CO_2_ selectivity and separation efficiency more effectively than continuous plasma or no plasma treatment, particularly at an equimolar feed ratio, as presented in Figure 2a,b.

Furthermore, at a feed composition of 92:8 (CH_4_:CO_2_), CO_2_ permeance remained relatively similar across the different operation modes, with values ranging from 1.3 × 10^−11^ to 1.6 × 10^−11^ (mol)/(m^2^·s·Pa), while CH_4_ permeance showed a slight reduction with pulsed plasma. Selectivity was consistently low (0.07–0.12), indicating a substandard separation performance under these conditions. In the literature, previous studies conducted separations of binary mixtures with an equimolar feed of CH_4_: CO_2_ (50:50). However, the presented work demonstrated the effect of separation with CH_4_:CO_2_ at 92:8 and 50:50. Considering the sub-atmospheric conditions, based on Figure 2c,d, it is observed that the pulsed plasma displayed higher performance compared to other modes of operation. Based on Figure S4a,b and Table S3, it is evident that most separation processes and membranes require high pressures for better performances; however, to overcome the challenges of separation at sub/atmospheric pressures, based on the four Rs of decarbonization (Reduce, Replace, Recycle, Remove), we need to re-design the process for effective separations, as demonstrated in this work. The development of novel/advanced porous materials for CO_2_ capture may address sector-specific challenges in separations with direct air capture [56,57,58,59]. With the current goal of decarbonization, industries have been considering integrating plasma-based processes [60].

### 3.2. Electrical Characterization

**Figure 3:**
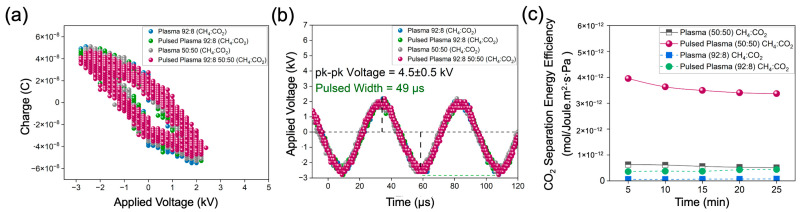
Electrical characterization during plasma operations with total flow rate of 200 sccm and feed ratio of 92:8 and equimolar ratio of 50:50 (CH_4_:CO_2_). (**a**) Lissajous curve using V-Q waveform for various membranes; (**b**) time vs. applied voltage for various membranes for calculating pk-pk voltage and frequency (kHz); (**c**) CO_2_ separation energy efficiency (mol)/(Joule·cm^2^·s·Pa) for plasma-based operations at different feed ratios.

**Figure 4:**
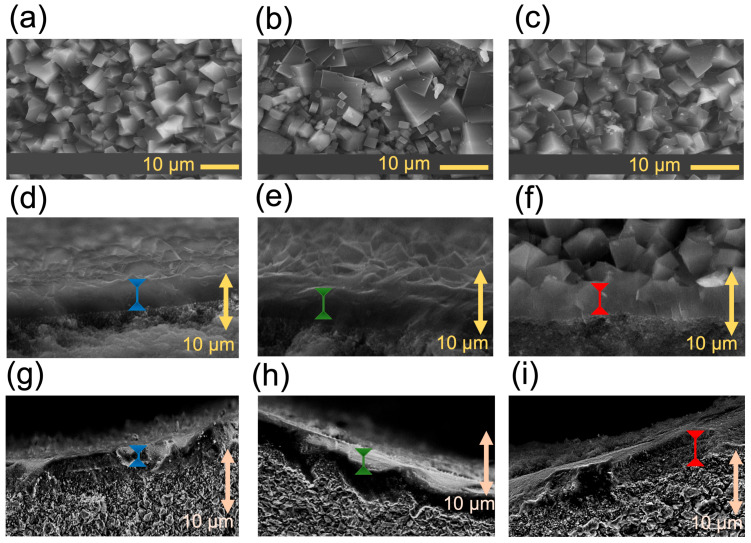
Scanning electron microscopy (SEM) images of microporous crystals employed as membranes in this study. (**a**) Fresh SAPO-34 membrane; (**b**) plasma-exposed SAPO-34 membrane; (**c**) pulsed-plasma-exposed SAPO-34 membrane; (**d**) cross-section of fresh SAPO-34 membrane; (**e**) cross-section of plasma-exposed membrane (92:8 CH_4_:CO_2_); (**f**) cross-section of pulsed-plasma-exposed SAPO-34 membrane (92:8 CH_4_:CO_2_); (**g**) cross-section of fresh SAPO-34 membrane; (**h**) cross-section of plasma-exposed membrane (50:50 CH_4_:CO_2_); (**i**) cross-section of pulsed-plasma-exposed SAPO-34 membrane (50:50 CH_4_:CO_2_).

The plasma operations were tailored for minimal/lower power settings, with the average power determined by calculating the area under the V-Q Lissajous plot curve and multiplying it by the frequency. Our major focus in conducting electrical characterization was directed toward understanding the electrical behavior and dielectric properties of membranes under mild plasma conditions. In Figure 3a, the voltage-charge characteristics of the DBD reactor during plasma operations (lasting 25 min) are depicted, conducted for 50:50 (CH_4_:CO_2_) and 92:8 (CH_4_:CO_2_) feed ratios and a 200 sccm total flow rate. It is important to note that the pressure outside the membrane (permeate) was <0.2 Psi during electrical data collection. The average power measured was 0.60 W, with a standard deviation of 0.09 W. The average plasma power (Watts) was maintained consistently for both feed ratios for an objective comparison of the performances. Meanwhile, Figure 3b illustrates the calculated frequency (1/time) at around 20.4 kHz and a pk-pk kV of 4.5 ± 0.5 kV. Interestingly, the separation efficiency saw an increase in the pulsed plasma mode compared to constant plasma. This was observed due to the lower input energy required for the pulsed mode—0.6 Watts for a total of 5 min—in contrast to constant plasma, which used 0.6 Watts for 25 min. By lowering the exposure time, the operation efficiency was increased (see Figure 3c). When comparing plasma to pulsed plasma, it became evident that constant plasma operations did not yield higher separation performance compared to pulsed plasma and also no plasma. We calculated energy efficiency as (CO_2_ Permeated)/(Energy Consumed) based on the energy input (Total Energy (Joules) = Power(W) × Time(s)). Based on the experimental data for pulsed plasma, the energy efficiency was 3.23 × 10^−12^ (mol)/(J.m^2^·s·Pa), which was 6.2 times higher than constant plasma (5.3 × 10^−13^ (mol)/(J.m^2^·s·Pa) (see Figure 4c). Comparing the feed ratios for separation efficiency, it is evident that the equimolar feed (50:50 CH_4_:CO_2_) with similar partial pressure led to higher separation efficiency compared to methane-rich feed (92:8 CH_4_:CO_2_). This observation could possibly be beneficial in the integration of intermittent technologies for renewable energy (solar or wind) generation.

### 3.3. Effect on Morphology of SAPO-34 Membranes

The SEM images in Figure 4 illustrate the impact of morphology in the absence and presence of plasma. In Figure 4a, the crystal size of fresh SAPO-34 membranes (pristine) is 5.3 ± 2.1 μm, while Figure 4d depicts the cross-section displaying a membrane thickness of 5.8 ± 1.2 μm. Interestingly, plasma exposure resulted in bimodal crystal sizes, with large crystals with sizes of 9.7 ± 2.1 μm and small crystals with sizes of 2.7 ± 1.8 μm in Figure 4b. Additionally, Figure 4e exhibits a cross-section with a membrane thickness of 4.7 ± 1.9 μm. Conversely, pulsed plasma exposure revealed crystal sizes of 3.7 ± 1.5 μm (Figure 4c), with Figure 4f displaying a cross-section exhibiting a membrane thickness of 5.2 ± 1.3 μm. Notable morphological changes were observed between constant plasma and pulsed plasma modes. Constant plasma exposure resulted in a bimodal crystal distribution in SAPO-34 (9.7 ± 2.1 µm and 2.7 ± 1.8 µm), while short doses of pulsed plasma produced a unimodal crystal size of 3.7 ± 1.5 µm. Our group has previously observed similar morphological changes in materials such as MOFs [61] and perovskites [62] after plasma exposure. By correlating these findings with our current work, we can leverage the advantages of pulsed plasma for short exposure times and efficient separation at sub-atmospheric pressures (please see Table 2). Furthermore, Figure 4g shows a cross-section with a membrane thickness of 4.7 ± 1.9 μm for pristine SAPO-34 membrane. Conversely, the equimolar feed ratio (CH_4_:CO_2_ 50:50) plasma exposure revealed a membrane thickness of 3.3 ± 0.9 μm (see Figure 4h), and in Figure 4i, the pulsed plasma exposure displays a cross-section exhibiting a membrane thickness of 4.6 ± 1.8 μm.

**Figure 5:**
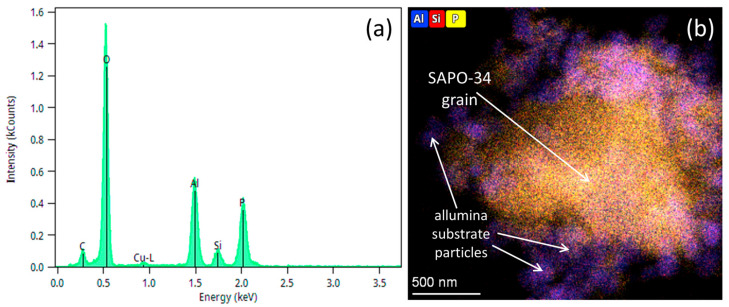
(**a**) Typical EDS spectrum obtained from the SAPO-34 membrane region. (**b**) Composite Al-Si-P elemental map from a SAPO-34 grain. Several small alumina substrate particles are shown.

We conducted XPS on pristine SAPO-34, constant plasma SAPO-34 and pulsed plasma SAPO-34 exposed membranes. The adventitious C1s peak, C-C at 284.5 eV, was utilized for the binding energy (BE) calibration. The values of BE obtained for Al2p, Si2p, P2p and O1s peaks for all three samples are listed in Table S1. Utilizing atomic sensitivity factors (ASFs), XPS data were used to evaluate the surface composition of these samples. The quantification values obtained from this analysis are shown in Table S2. Notice the presence of additional small particles originating from the alumina support (see Figure S6) showing typical EDS elemental maps obtained for all three samples. The corresponding STEM-HAADF images (Column 1) and EDS spectra (Column 7) are also included in Figure S6. These data indicate that both the elemental composition and morphology of SAPO-34 grains are consistent between these three samples. A typical EDS spectrum obtained from the membrane material is shown in Figure 5a.

**Figure 6:**
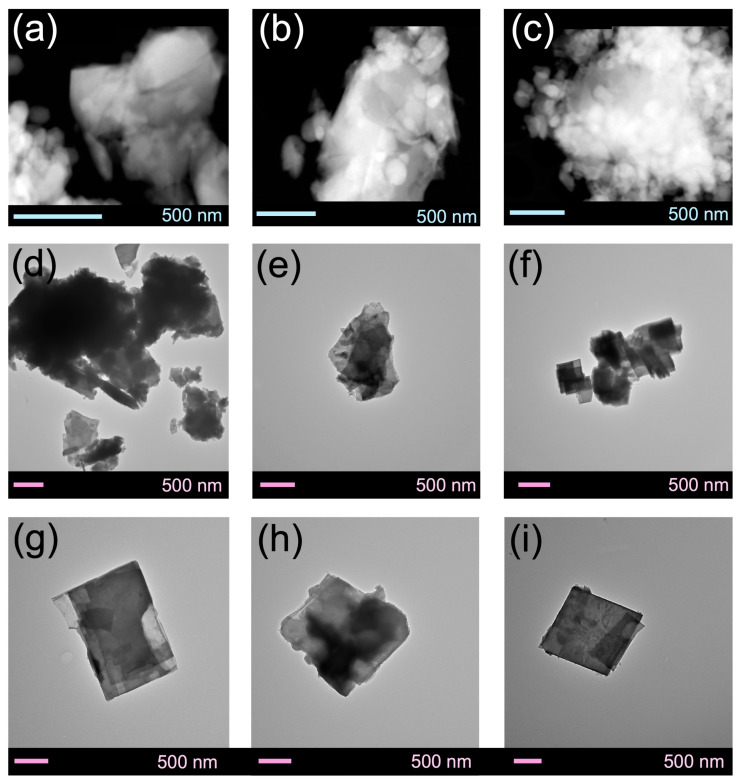
STEM-HAADF images (first row) of (**a**) SAPO-34-Fresh, (**b**) SAPO-34-Constant Plasma and (**c**) SAPO-34-Pulsed Plasma in methane-rich feed (92:8 CH_4_:CO_2_); (second row) TEM (**d**) SAPO-34-Fresh, (**e**) SAPO-34-Constant Plasma and (**f**) SAPO-34-Pulsed Plasma in equimolar feed (50:50 CH_4_:CO_2_); (third row) close-up view of (**g**) SAPO-34-Fresh, (**h**) SAPO-34-Constant Plasma and (**i**) SAPO-34-Pulsed Plasma in equimolar feed (50:50 CH_4_:CO_2_).

The quantification analysis of these data yields concentrations of 15.8 atm%, 3.2 atm%, 12.3 atm% and 68.7 atm% for Al, Si, P and O, respectively. Compared to the surface concentrations obtained from XPS analysis, these values are much closer to the nominal concentrations of 14.5 atm%, 4.3 atm%, 14.5 atm% and 66.7 atm% expected for SAPO-34 described by the chemical formula Al_10_Si_3_P_10_O_46_ (i.e., 5Al_2_O_3_:5P_2_O_5_:3SiO_2_). The discrepancy between EDS and XPS quantification results originates from the fact that XPS is a surface technique probing only 1–2 nm of the subsurface layer, while EDS provides a signal from much thicker layers, often in the hundreds of nanometers or sub-micrometer-thickness range. EDS elemental maps were also collected from these membrane samples, confirming the uniform distribution of Al, Si, P and O across SAPO-34 grains, as shown in Figure S6 in a typical composite Al-Si-P map obtained for these samples. Similar observations can be made in terms of the effect on morphology with different feed ratios. Based on the experimental observations and TEM images, constant plasma exposure resulted in significant chemical and structural modifications (see Figure 6b,e,h).

Based on the experimental data, the results from XPS revealed that more surface alterations were observed with constant plasma, while minor changes in surface composition were detected after pulsed plasma exposure. The decrease in O1s BE values suggests a reduction in the oxidation state of oxygen species (Fresh (532.2 eV) > Pulsed Plasma (532 eV) > Plasma (531.9 eV)). The feed composition facilitated important insights on membrane surface, as the 92:8 (CH_4_:CO_2_) feed had a higher presence of hydrogen atoms and fewer oxygen atoms, creating a reduced environment. Based on the binding energies, the CH_4_-rich (92:8) feed composition created a reduced environment, leading to lower BE values for Si2p and O1s, indicating a shift to lower oxidation states. Hence, the alteration of surface composition can be directly correlated with feed composition of the gas on the membrane, and this information may aid in designing future methods to tailor membrane composition for its longevity, multiple cycles and efficient separation.

**Figure 7:**
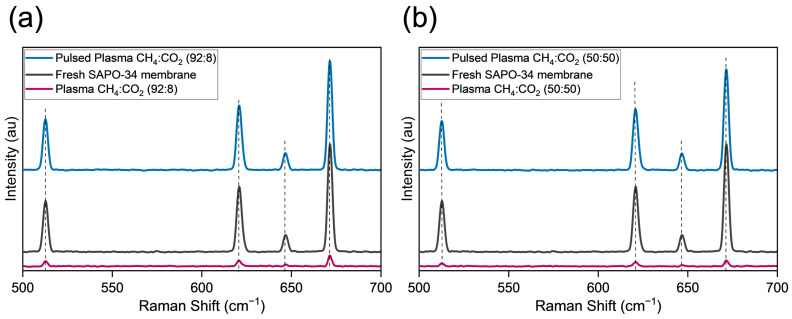
Comparative analysis using Raman spectroscopy: (**a**) methane-rich feed (92:8) CH_4_:CO_2_; (**b**) equimolar feed (50:50) CH_4_:CO_2_ (note: *Y*-axis is similar in both plots for homogeneity).

In order to gain more insights into the membranes’ molecular compositions and their interactions, Raman spectroscopy was conducted (please see Figure 7). Based on the data, the intensity of exposed plasma and pulsed plasma was compared with fresh SAPO-34 membranes. Since membranes are composite materials (SAPO-34 crystals on a porous alumina substrate), the analysis can be arduous in terms of reproducibility. Spectroscopy was conducted at various locations within the membranes and further averaged in triplicate. Comparing the intensity with that of a fresh SAPO-34 membrane, we observe a drastic change. For the plasma-exposed membrane, the intensity was reduced on average, while for the membrane exposed to pulsed plasma, the intensity increased. Significantly, the bands observed at 621.16 cm⁻^1^, 646.79 cm⁻^1^ and 671.29 cm⁻^1^ fall within the range of 400–650 cm⁻^1^, which is associated with bending vibration of the bridging oxygen (BO) bonds in SiO₄ tetrahedra [63]. On the other hand, at 513.25 cm⁻^1^, the peak is in the range of 450–600 cm⁻^1^, which is related to motions of bridged oxygen in T-O-T association (wherein T can be Si or Al) [63]. When employing pulsed plasma, we observed improved crystallinity, which could be correlated with SEM and XPS data. This trend was also consistent with our previous observations in various materials exposed to plasma, including inorganic materials (SAPOs, perovskites) [46,62,64,65,66], hybrid materials (metal–organic frameworks) [41,61,67] and organic materials (porous organic cages) [52].

### 3.4. Plausible Understanding of CO_2_/CH_4_ Plasma-Mediated Separations

**Figure 8:**
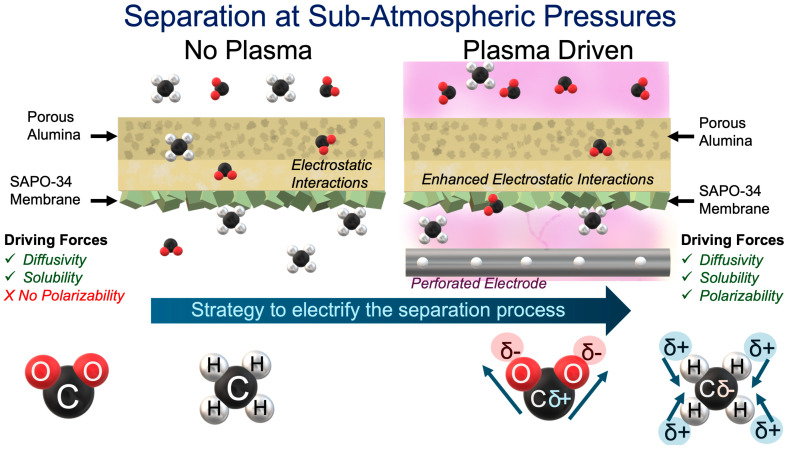
Plausible mechanistic insight on CO_2_/CH_4_ separation at sub-atmospheric pressures.

The SAPO-34 membranes reported here displayed higher performance when submitted to an intermittent plasma exposure; such experimental conditions could enhance gas separation through different pathways. Regarding the plasma effect on the membrane, it has been reported that defects and cracks in zeolite membranes, often considered undesirable, can actually play a crucial role in enhancing gas selectivity. While the uniform, molecular-sized pores of zeolites make them ideal candidates for separation processes, the presence of defects—larger intercrystalline spaces—can introduce new pathways for selective permeation [68]. These defects can possibly change due to temperature variations and can alter the diffusion and adsorption behavior of gases within the membrane. This flexibility in the membrane structure allows for selective gas transport based on differences in molecular size and adsorption strength. Intermittent plasma exposure might generate a network of controlled defects on the membrane surface, developing microcracks that, based on observations from the literature [69,70], improve the mechanical properties while enabling new preferential transport channels for selective gas molecules. When analyzing the effect of intermittent plasma conditions on the gas phase, we have to focus on the chemistry of polar molecules in the presence of an electric field, wherein the electron clouds of these CO_2_ molecules can become distorted, inducing temporary dipoles (see Figure 8). It is important to note that in the absence of an electric field, both CO_2_ and CH_4_ are non-polar molecules exhibiting 0.00 Debye Å (zero dipole moments) [59]; however, plasma could lead to controllable interactions when looking at polarizabilities and quadrupole moments that could lead to different behaviors in the absence and presence of an electric field. First, in terms of the molecular structure, methane’s tetrahedral symmetry makes it thermodynamically stable and a challenge to dissociate or separate [71,72]. In contrast, the linear structure of CO_2_ allows it to be easily polarized, facilitating its separation. This distortion of electron clouds in response to the electric field is known as polarizability. In the case of our membrane (~SAPO-34), in the literature, it is well noted that the intermolecular forces play an integral role in exhibiting strong electrostatic interactions [73]. With greenhouse gases such as CO_2_ and CH_4_, we can observe that polarizability is somewhat easier to achieve in CO_2_ (2.9Å^3^) > CH_4_ (2.6Å^3^) [74]. Thus, the enhancement in electrostatic interactions can be correlated with improved separation in the pulsed plasma and plasma modes of operation, as molecules with higher polarizability (~CO_2_) might experience more significant distortion and thus exhibit stronger electrostatic interactions with each other and with the applied field. Another argument to reinforce these findings is to compare the electronegativity. As in the case of an electric field, there may be a shift in the quantitative value of electron density, but the electronegativity will remain the same: Oxygen (3.4) > Carbon (2.5) > Hydrogen (2.2).

The electron affinity values of CO_2_ (−0.51 eV) [75] and CH_4_ (+0.19 eV) [75] suggest that CO_2_ has a higher favorability to accept electrons compared to CH_4_. On the other hand, CO_2_ has a quadrupole moment [76] due to its linear structure, with partial positive and negative charges at different points. This allows CO_2_ to interact electrostatically with the polar surface of SAPO-34. While CH_4_ is a non-polar molecule, it can still interact with the zeolite surface through Van der Waals forces [77], which are weaker than electrostatic interactions. The SAPO-34 framework may also have acidic sites—specifically, hydroxyl groups [78,79]—that may form weak hydrogen bonds or dipole interactions with CO_2_. CO_2_ molecules can interact with the framework’s aluminum and silicon atoms through their quadrupole moments, which align with the electric fields generated by the framework’s charge distribution. The difference in interaction strengths can lead to selective adsorption, where CO_2_ is preferentially adsorbed over CH_4_ due to stronger electrostatic interactions, enhancing the separation process in materials like SAPO-34. The tailored arrangement atoms (~Si, Al, P) in SAPO-34 may influence the extent of these interactions [46], with the polarizability of the framework playing a critical role. In our previous work, we observed the importance of plasma and zeolite-cage synergism, specifically with SAPO-34, providing enhanced CO_2_ adsorption possibly due to the CHA structure increasing the magnitude of CO_2_–CO_2_ interactions. This can be envisioned as two CO_2_ molecules strongly adsorbed on opposite pore surfaces exerting greater adsorption influence on a third CO_2_ molecule positioned between them [46]. More details on the comparative properties of CH_4_ and CO_2_ are provided in Table S4.

## 4. Conclusions

In conclusion, the electrification of gas separation is critical for enhancing sustainability and substituting high-energy-intensive membrane technologies. Traditional membrane methods, reliant on high pressures, are inherently energy-demanding. The CO_2_/CH_4_ separation experiments in this paper involved no plasma, constant plasma and pulsed plasma modes. The pulsed plasma mode showed the highest CO_2_ permeance and selectivity (~1.8) at an equimolar (50:50) feed ratio, with performance declining over time, aligning with trends found in the literature. At a 92:8 (CH_4_:CO_2_) feed ratio, CO_2_ permeance remained similar across modes, but separation of CO_2_ was poor due to the low partial pressure. Overall, pulsed plasma enhanced the separation efficiency under sub-atmospheric conditions. By introducing the potential of separating gases such as CO_2_ and CH_4_ using low-dose plasma at sub-atmospheric pressure, this research presents a more energy-efficient alternative. In future studies, it would be interesting to explore polarizability with different gas pairs in binary, ternary and multi-component modes to validate its applicability in gas separation (examples: CH_4_ (2.6Å^3^), CO_2_ (2.9Å^3^), O_2_ (1.58Å^3^), CO (1.95Å^3^), H_2_ (0.80Å^3^), N_2_ (1.74Å^3^), NH_3_ (2.81Å^3^), Kr (2.48Å^3^) and Xe (4.04Å^3^)). This innovative approach significantly reduces the environmental impact, paving the process for a more sustainable future.

## Figures and Tables

**Table 1:** Comparative analysis of CO_2_/CH_4_ separation with and without plasma at 25 min.

Operation	Feed Ratio	CO_2_ Permeance	CH_4_ Permeance	Selectivity (α)	Permeate Pressure	Separation Index
	CH_4_:CO_2_	(mol)/(m^2^·s·Pa)	(mol)/(m^2^·s·Pa)	CO_2_/CH_4_	Pascal	π
No Plasma	92:8	1.3 × 10^−11^	1.7 × 10^−10^	0.08	1378	−1.6 × 10^−8^
No Plasma	50:50	1.1 × 10^−10^	9.0 × 10^−11^	1.25	1378	3.9 × 10^−8^
Plasma	92:8	1.4 × 10^−11^	1.9 × 10^−10^	0.07	1378	−1.8 × 10^−8^
Plasma	50:50	1.0 × 10^−10^	7.8 × 10^−11^	1.29	1378	3.9 × 10^−8^
Pulsed Plasma	92:8	1.6 × 10^−11^	1.3 × 10^−10^	0.12	1378	−1.9 × 10^−8^
Pulsed Plasma	50:50	1.2 × 10^−10^	6.6 × 10^−11^	1.81	1378	1.3 × 10^−7^

Note: Separation index π ((CO_2_ permeance × (selectivity − 1)) × permeate pressure).

**Table 2:** SAPO-34 membrane crystal size distributions and cross-sectional thicknesses.

Membrane	Average Crystal Size		Average Thickness (92:8 CH_4_:CO_2_)	Average Thickness (50:50 CH_4_:CO_2_)
	μm		μm	μm
SAPO-34 Fresh	5.3 ± 2.1		5.8 ± 1.2	2.4 ± 1.8
SAPO-34 Plasma	9.7 ± 2.1	2.7 ± 1.8	4.7 ± 1.9	3.3 ± 0.9
SAPO-34 Pulsed Plasma	3.7 ± 1.5		5.2 ± 1.3	4.6 ± 1.8

## Data Availability

The original contributions presented in the study are included in the article and Supplementary Materials, further inquiries can be directed to the corresponding author.

## Supplementary Materials

The following supporting information can be downloaded at: https://www.mdpi.com/article/10.3390/membranes14080178/s1, Figure S1: Membrane continuity testing with 1.8% propane/nitrogen mixtures; Table S1: Binding energy values obtained from XPS analysis; Table S2: XPS-based elemental quantification without carbon; Figure S2: Non-thermal plasma separation unit was employed in this study; Figure S3: Scanning electron microscopy image of membrane and porous substrate interface; Figure S4: State-of-the-art comparison; Table S3: State-of-the-art CO_2_/CH_4_ Separation via membranes; Figure S5: Breakthrough experiments with equimolar (50:50) mixtures of CH_4_/N_2_ gas over SAPO-34 membrane; Figure S6: TEM-HAADF-EDS mapping; Table S4: Important properties of greenhouse gases (GHGs) presented in this work. References [9,59,74,75,80,81] are cited in Supplementary Materials.
