# Supplementary material for: Performance and Enhanced Efficiency Induced by Cold Plasma on SAPO-34 Membranes for CO2 and CH4 Mixtures
Source: Membranes (Basel). 2024 Aug 20;14(8):178. doi: 10.3390/membranes14080178 (PMC11356667; doi:10.3390/membranes14080178)
Supplement: Supplementary file 1 [file membranes-14-00178-s001.zip › membranes-3144531-supplementary.pdf]

membranes

MDPI

Article

Performance and Enhanced Efficiency Induced by Cold Plasma on SAPO-34 Membranes for CO_2 and CH_4 Mixtures

FNU Gorky [1], Vashanti Storr [1], Grace Jones [1], Apolo Nambo [2], Jacek B. Jasinski [3] and Maria L. Carreon [1,*]

[1] Ralph E. Martin Department of Chemical Engineering, University of Arkansas, Fayetteville, AR 72701, USA; gorky@uark.edu (F.G.); vstorr@uark.edu (V.S.); gej006@uark.edu (G.J.)
[2] Bert Thin Films, LLC., 625 Myrtle St, Louisville, KY 40298, USA; apolo@bertthinfilms.com
[3] Conn Center for Renewable Energy Research, University of Louisville, Louisville, KY 40292, USA; jacek.jasinski@louisville.edu
* Correspondence: mc138@uark.edu

Membrane Continuity Testing

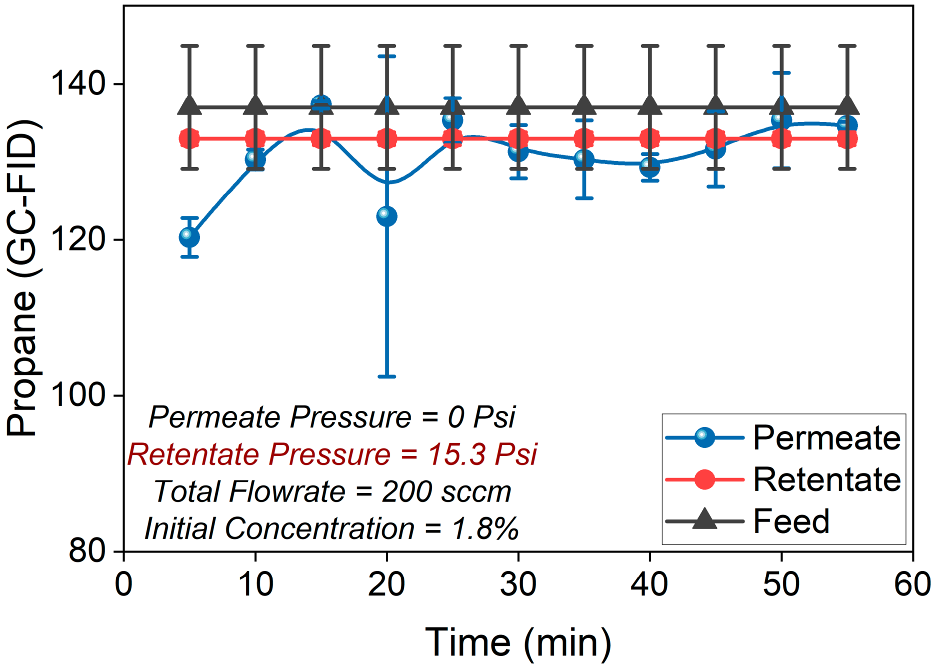

Figure S1. Membrane continuity testing with 1.8% Propane/Nitrogen mixtures.

XPS data

Table S1. BE values obtained from XPS analysis for SAPO-34 interaction with 92:8 (CH_4:CO_2) and 50:50 (CH_4:CO_2) feed composition.

	**Feed Composition 92:8(CH_4:CO_2)**			
	**Al2p (eV)**	**Si2p (eV)**	**P2p (eV)**	**O1s (eV)**
**SAPO-34-Fresh**	74.8	103.0, 100.1	133.9	532.2
**SAPO-34-Pulsed Plasma**	74.6	100.2	133.9	532.0
**SAPO-34-Plasma**	74.6	99.7	133.9	531.9
	Feed Composition 50:50 (CH_4:CO_2)			
	**Al2p (eV)**	**Si2p (eV)**	**P2p (eV)**	**O1s (eV)**
**SAPO-34-Fresh**	74.0	101.7	133.2	531.0
**SAPO-34-Pulsed Plasma**	72.7	100.7	132.7	530.2

	**Feed Composition 92:8(CH_4:CO_2).**			
92:8(CH_4:CO_2)	**Al (atm%)**	**Si (atm%)**	**P (atm%)**	**O (atm%)**
**SAPO-34-Fresh**	7.3	16.5	10.4	65.8
**SAPO-34-Pulsed Plasma**	8.9	17.8	9.3	64.0
**SAPO-34-Plasma**	9.5	18.1	10.5	62.0
	Feed Composition 50:50 (CH_4:CO_2)			
50:50 (CH_4:CO_2)	**Al (atm%)**	**Si (atm%)**	**P (atm%)**	**O (atm%)**
**SAPO-34-Fresh**	8.9	6.5	11.7	62.9
**SAPO-34-Pulsed Plasma**	7.4	4.7	10.1	49.5

Table S2. XPS-based elemental quantification without carbon.

	**Al (atm%)**	**Si (atm%)**	**P (atm%)**	**O (atm%)**
**Fresh**	7.3	16.5	10.4	65.8
**Exposed 5 min**	8.9	17.8	9.3	64.0
**Exposed 10 minutes**	9.5	18.1	10.5	62.0

Non-Thermal Plasma-Separation Unit

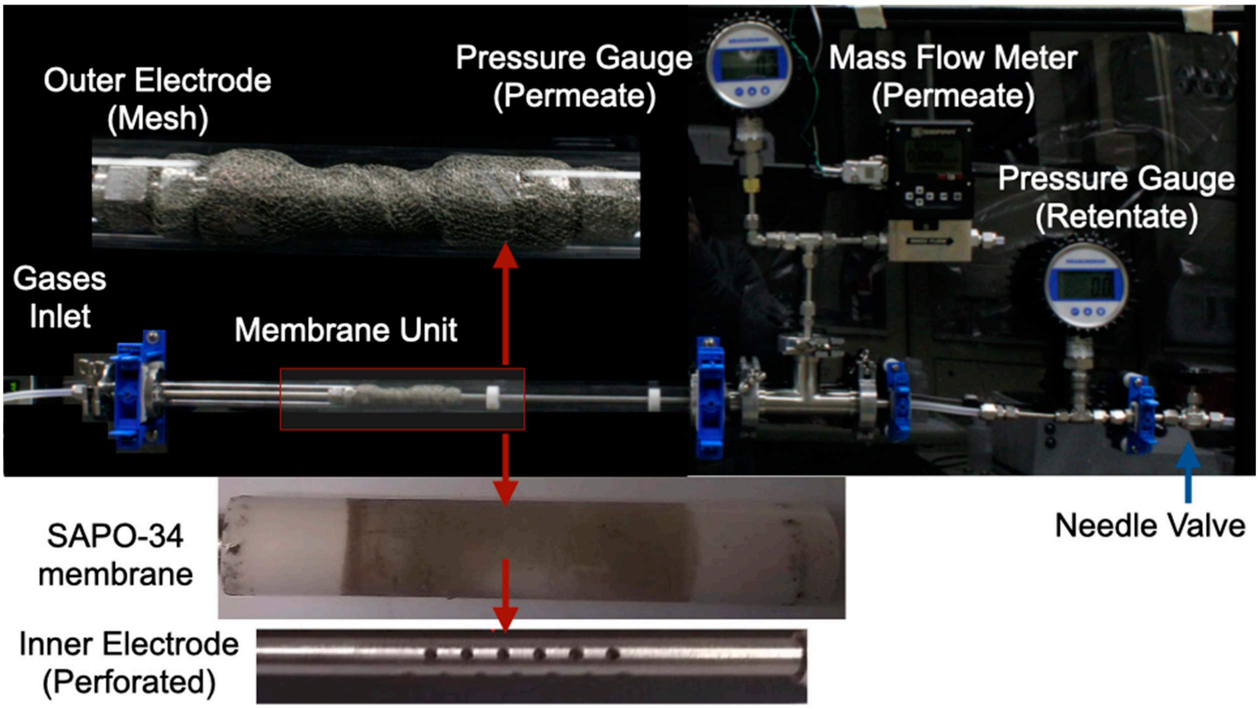

Figure S2. Non-Thermal Plasma Separation unit was employed in this study.

SEM image (Membrane and Porous Substrate interface)

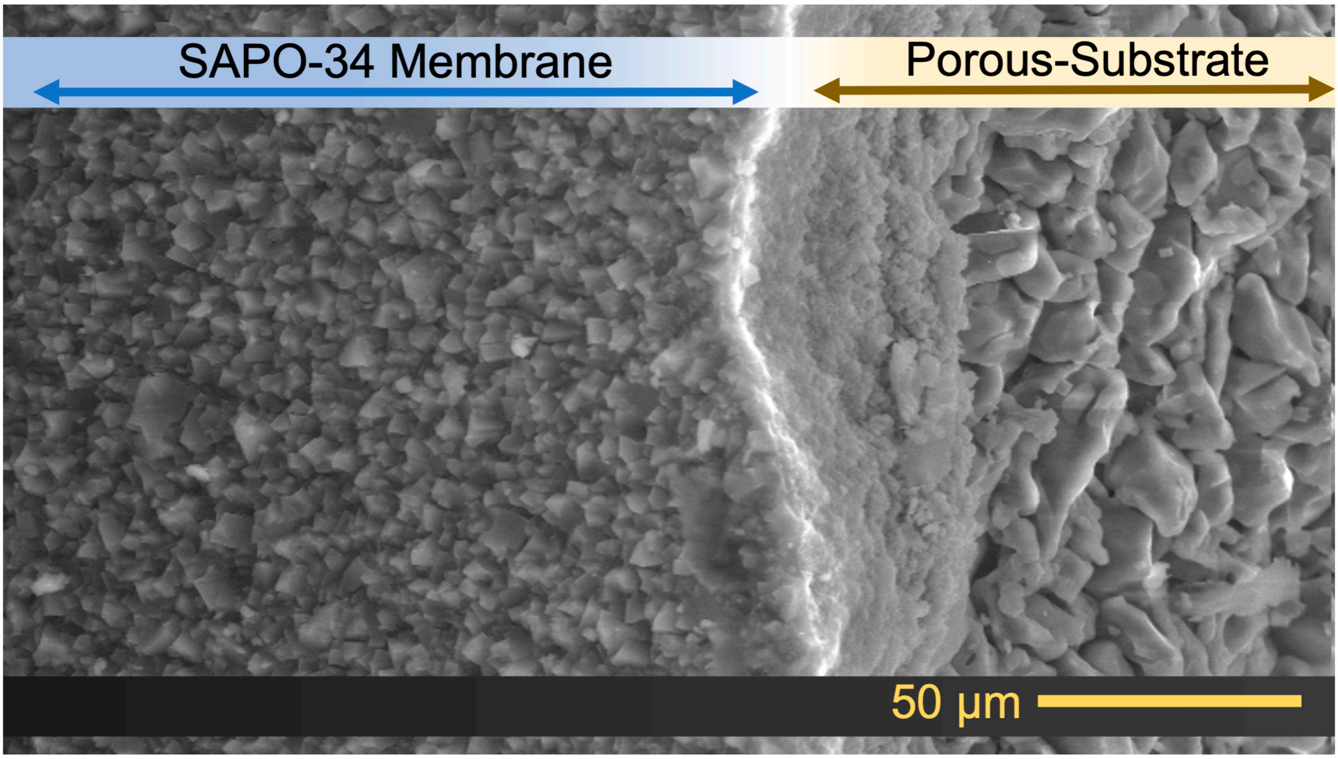

Figure S3. Scanning Electron Microscopy image of membrane and porous substrate interface.

CH_4/CO_2 Membrane Separation State of the Art

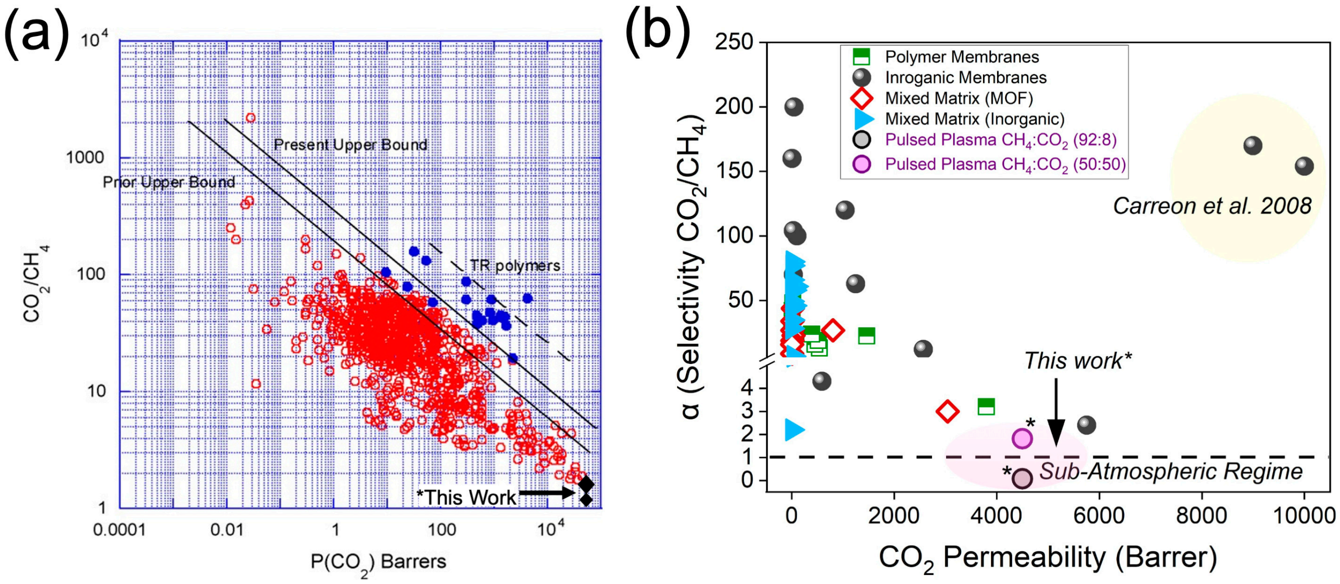

Figure S4. (**a**) Modified Robeson plot for CO_2/CH_4 separations representing the upper bound; (**b**) State-of-the-art figure displaying performance for various types of membranes in CO_2/CH_4 separations.

State-of-the-art table

Table S3. State-of-the-art CO_2/CH_4 Separation via membranes.

Membrane	Molar Feed Ratio	Temperature	CO_2 permeance	Selectivity	Separation Index (π)	Ref.
	(CO_2:CH_4)	°C	×10−8(mol/m²·s·Pa)	(CO_2/CH_4)	e×10⁻²(mol/m²·s)	
SAPO-34 A1	50:50	25	1×10^6	86	7.4	[1]
SAPO-34 A2	50:50	25	1×10^6	99	8.6	
SAPO-34 A3	50:50	25	1.2×10^6	131	13	
SAPO-34 A4	50:50	25	2×10^6	86	14	
SAPO-34 A5	50:50	25	1.8×10^6	171	25	
SAPO-34 nonfunctionalized	50:50	25	4.6×10^7	159	7.3	[2]
SAPO-34 0.15 mmol of ED	50:50	25	4.9×10^7	245	12	
SAPO-34 0.33 mmol of ED	50:50	25	4.4×10^7	233	10.3	
SAPO-34 1.66 mmol of ED	50:50	25	4.4×10^7	164	7.1	
SAPO-34 8.32 mmol of ED	50:50	25	5.3×10^7	92	4.8	
SAPO-34 0.15 mmol of HA	50:50	25	3.7×10^7	238	8.72	
SAPO-34 0.15 mmol of OA	50:50	25	1.9×10^7	229	4.31	
M1,2,3- 25 cm	50:50	25	6.6×10^7	142	7.8	[3]
M4,5,6- 25 cm	50:50	25	4.9×10^7	214	8.8	
M7,8,9- 5 cm	50:50	25	5.6×10^7	234	10.8	
M10,11,12- 25 cm	50:50	25	2.5×10^7	248	5.1	
M13-M18 - 5 cm	50:50	25	4.4×10^7	256	9.3	
M19,20 – 5 cm	50:50	25	4.5×10^7	254	9.6	
Notes:						

Breakthrough Curve

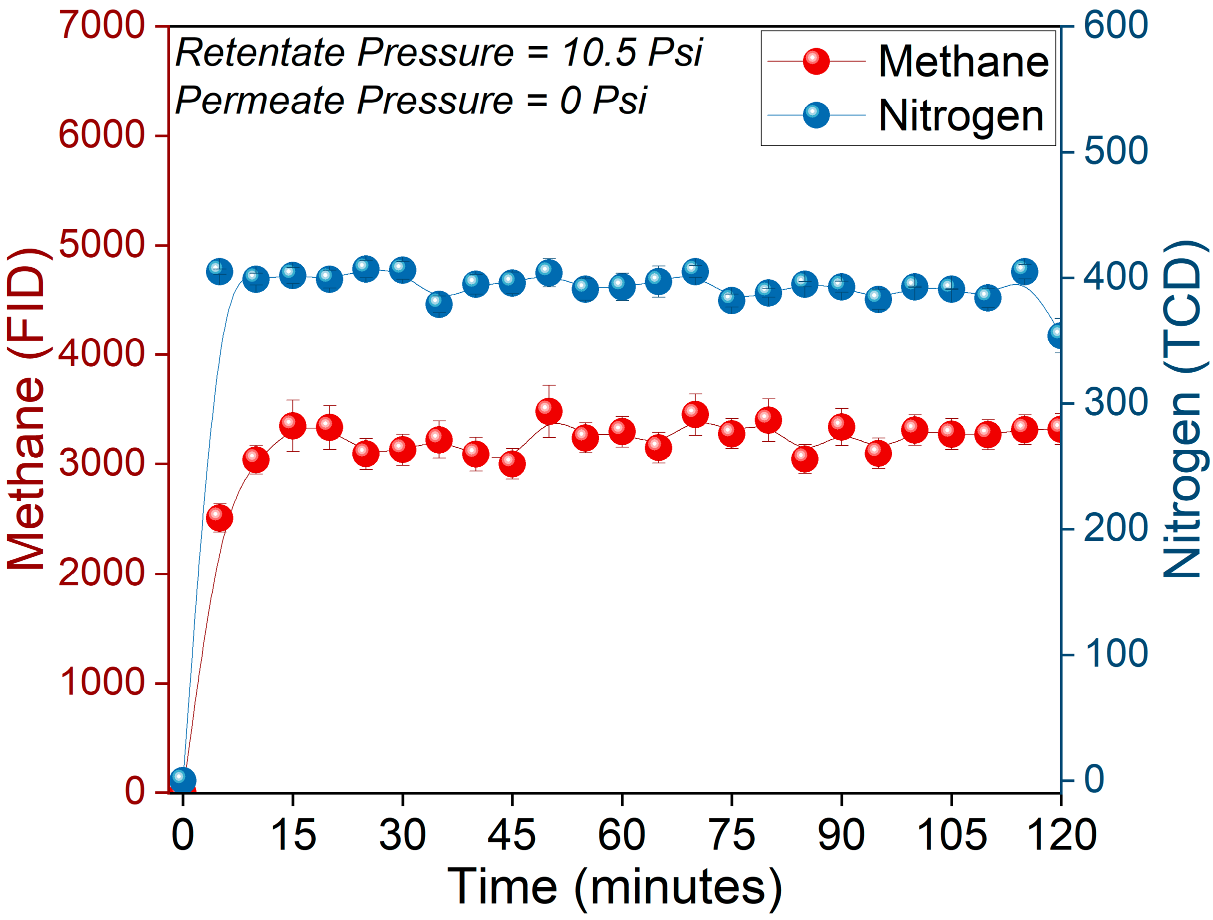

Figure S5. Breakthrough experiments with equimolar (50:50) mixtures of CH_4/N_2 gas over SAPO-34 membrane.

TEM-HAADF EDS Mapping

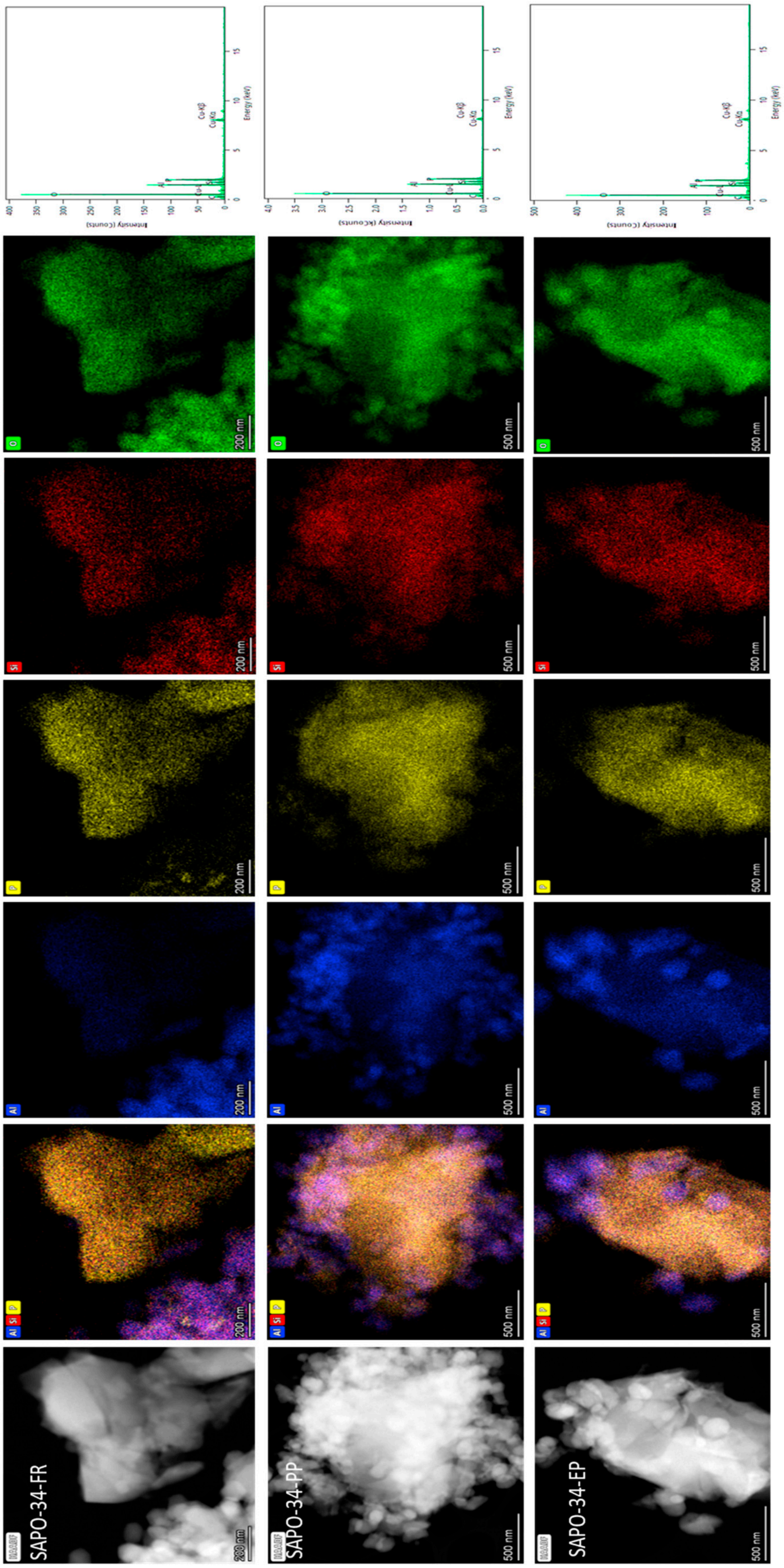

Figure S6. STEM-HAADF images (1st column), elemental maps (columns 2-6), and EDS spectra (column 7) of SAPO-34-FR (top), SAPO-34-PP (middle), and SAPO-34-EP (bottom) samples.

Table S4. Important Properties for presented Greenhouse Gases (GHGs) in this work.

Property		Units	Carbon Dioxide	Methane
**Physical**	Molecular Weight	g/mol	44.01	16.04
	Boiling Point	°C	-78.5	-161.5
	Melting Point	°C	-56.6	-182.5
	Freezing Point	°C	-78.5	-182.5
	Density (Gas, STP)	g/L	1.98	0.717
	Heat Capacity (Gas, STP)	J/mol·K	37.1	35.7
	Critical Temperature	°C	31.0	-82.6
	Critical Pressure	atm	73.8	45.8
	Density (Gas, STP)	g/L	1.98	0.717
**Diffusivity**	Kinetic Diameter	Å	3.30	3.80
	Collision Diameter	Å	3.70	3.80
	Diffusion Coefficient	cm²/s	0.138	0.202
	Diffusivity in air	cm²/s	0.16	0.21
	Schmidt Number	-	0.97	0.61
**Thermodynamic**	ΔH_{vap}	J/mol·K At STP	37.1	35.7
**Electric Field**	Dipole Moment	Debye	0.00 [4]	0.00 [4]
	Quadrupole Moment	Debye Å	4.30 [4]	0.02 [4]
	Polarizability	Å³	2.9 [5]	2.5 [5]
	Dielectric Constant	-	1.000984	1.000918
	Refractive Index	-	1.00045	1.000444
	Ionization Energy	eV	13.77	12.61
	Bond Dissociation Energy	eV	5.5	4.7
	Electronic Affinity	eV	-0.51 [6]	+0.19 [6]
**Environmental**	GWP (Global Warming Potential)	20 year timescale	1	84
	Atmospheric Lifetime	years	50-200	12
	Radiative Efficiency	W/m²/ppb	1.3 e-5	3.6 e-4
	Typical Atmospheric Concentration	ppm	419	1.9
	Atmospheric Concentration Growth Rate	%/year	0.6	2.5
	Carbon Cycle Impact	-	Acts as a critical component with long-term storage capabilities in oceans and the terrestrial biosphere.	Displays a short atmospheric lifetime, subsequently oxidizing to CO2, thereby further contributing to the greenhouse effect.

References

1. Carreon, M.A.; Li, S.; Falconer, J.L.; Noble, R.D. Alumina-supported SAPO-34 membranes for CO_2/CH_4 separation. *J. Am. Chem. Soc.* **2008**, *130*, 5412–5413.
2. Venna, S.R.; Carreon, M.A. Amino-functionalized SAPO-34 membranes for CO2/CH4 and CO2/N2 separation. *Langmuir* **2011**, *27*, 2888–2894.
3. Li, S.; Carreon, M.A.; Zhang, Y.; Funke, H.H.; Noble, R.D.; Falconer, J.L. Scale-up of SAPO-34 membranes for CO2/CH4 separation. *J. Membr. Sci.* **2010**, *352*, 7–13.
4. Pera-Titus, M. Porous inorganic membranes for CO_2 capture: Present and prospects. *Chem. Rev.* **2014**, *114*, 1413–1492.
5. Miller, T.M. Atomic and molecular polarizabilities. *CRC Handb. Chem. Phys.* **2000**, *77*, 193–202.
6. Johnson, R.D., III. NIST 101. Computational chemistry comparison and benchmark database. **1999**. Available online: https://cccbdb.nist.gov/elecaff2x.asp?casno=124389 (accessed on 21 July 2024).
